# Do Certain Flavonoid IMPS Have a Vital Function?

**DOI:** 10.3389/fnut.2021.762753

**Published:** 2021-12-01

**Authors:** David S. Seigler, J. Brent Friesen, Jonathan Bisson, James G. Graham, Ana Bedran-Russo, James B. McAlpine, Guido F. Pauli

**Affiliations:** ^1^Department of Plant Biology, University of Illinois at Urbana Champaign, Champaign, IL, United States; ^2^Center for Natural Products Technologies, College of Pharmacy, University of Illinois at Chicago, Chicago, IL, United States; ^3^Pharmacognosy Institute, College of Pharmacy, University of Illinois at Chicago, Chicago, IL, United States; ^4^Department of Pharmaceutical Sciences, College of Pharmacy, University of Illinois at Chicago, Chicago, IL, United States; ^5^Physical Sciences Department, Dominican University, River Forest, IL, United States; ^6^Department of Restorative Dentistry, College of Dentistry, University of Illinois at Chicago, Chicago, IL, United States

**Keywords:** flavonoids, Vitamin P, Vitamin C, invalid metabolic panaceas (IMPS), vitamins, micronutrients, cofactors

## Abstract

Flavonoids are a vast group of metabolites that are essential for vascular plant physiology and, thus, occur ubiquitously in plant-based/-derived foods. The solitary designation of thousands of known flavonoids hides the fact that their metabolomes are structurally highly diverse, consist of disparate subgroups, yet undergo a certain degree of metabolic interconversion. Unsurprisingly, flavonoids have been an important theme in nutrition research. Already in the 1930s, it was discovered that the ability of synthetic Vitamin C to treat scurvy was inferior to that of plant extracts containing Vitamin C. Subsequent experimental evidence led to the proposal of Vitamin P (permeability) as an essential phytochemical nutrient. However, attempts to isolate and characterize Vitamin P gave confusing and sometimes irreproducible results, which today can be interpreted as rooted in the unrecognized (residual) complexity of the intervention materials. Over the years, primarily flavonoids (and some coumarins) were known as having Vitamin P-like activity. More recently, in a NAPRALERT meta-analysis, essentially all of these Vitamin P candidates were identified as IMPs (Invalid/Improbable/Interfering Metabolic Panaceas). While the historic inability to define a single compound and specific mode of action led to general skepticism about the Vitamin P proposition for “bioflavonoids,” the more logical conclusion is that several abundant and metabolically labile plant constituents fill this essential role in human nutrition at the interface of vitamins, cofactors, and micronutrients. Reviewing 100+ years of the multilingual Vitamin P and C literature provides the rationales for this conclusion and new perspectives for future research.

## Origin of This Study

The original motivation for this study came from the recent proof of the existence of invalid/improbable/interfering metabolic panaceas (IMPS) (1) as over-studied natural products that have an implausible plethora of reported biological activities, making them panaceas—in theory. At the same time IMPS are very commonly found in plant-derived food products, implying that IMPS play potential roles in human nutrition. Following the premise that unrecognized life-essential biological functions of molecules may potentially blur their experimentally observable *in vivo* and *in vitro* effects, we sought to unravel the link between flavonoids and Vitamin P (VitP). The commonly used synonym, “bioflavonoids,” already hints at the potentially blurred scientific standing of VitP. In contrast, flavonoids are well-defined phytochemicals that are nearly ubiquitous in plants and likely represent the most well-studied class of plant constituents. Interestingly, yet not surprisingly, flavonoids comprise the by far largest group in the top-38 known IMPS (1).

## Motivation of This Study

Building on more than a century of primary literature published in English, French, and German, the present study compiles existing evidence and presents an intriguing new view for the connection between the existence of Vitamin P (VitP) as a known, but difficult to identify, cofactor of vital body functions in humans, and flavonoid dietary plant constituents that have been designated as IMPS. The study develops a set of rationales for the ability of flavonoids and some phenylpropanoids, structurally and biogenetically closely related plant phenols, to possess biological functions that are essential for healthy human life. These functions involve peripheral collagenous, vascular tissue, and include the permeability of vascular capillaries and cerebral tissue. Accordingly, flavonoids and related compounds must impact cardiovascular and general human health significantly. Despite them being phenols, chemically, this study also brings about new rationales why their categorization as “polyphenols” has mostly confusing biochemical and biological implications that make this term problematic.

The surprisingly elusive nature and lack of an assigned single chemical entity of these, otherwise essential, bioactive molecules that constitute VitP may well be rationalized by the dietary omnipresence and metabolic interconversion of the flavonoid species, as they have been experimentally associated with both VitP and Vitamin C (VitC) bioactivities. This directly leads into the metabolomic complexity of plant phenols: Thousands of flavonoids and a somewhat smaller number of coumarins exist with the basic ring structure required for VitP activity. However, the activity and quantity of various flavonoids varies tremendously. The most commonly sold, rutin, has very little activity, whereas epicatechin is reported to have much greater activity.

## Overcoming Facile Structure-Bioactivity Paradigms

The observation that P is the last letter in the historic “vitamin alphabet” (Table 1) which has not been linked to a triplet of a strictly deficient diet, biochemical processes, and well-defined chemistry indicates why the scientific community has hesitated to assign VitP the role of a vitamin, cofactor, or micronutrient. To achieve a new overall perspective, this study took a two-fold approach by (A) collating existing knowledge via a comprehensive review of 100+ years of relevant literature; and (B) evaluating the findings with respect to contemporary knowledge in phytochemistry, biochemistry, and biology.

**Table 1:** The alphabet of vitamins, their chemical description, deficiency conditions or diseases, and basic discovery facts.

**Vitamin**	**Chemistry**	**Deficiency**	**Brief discovery facts**
A	Retinol	Visual impairment (among others)	Magendie 1816; Hopkins 1912 (Nobel Prize 1929) from milk, McColumn and Davis and Mendel and Osborne 1913; chemical structure by Karrer 1913
B_1_	Thiamine	Beriberi	Kanehiro 1884 germ theory rejection, Williams 1934 structure elucidation; mainly from rice bran
B_2_	Riboflavin	Stomatitis (among others)	Kuhn, György, and Wagner 1933–1939 from egg white and whey
B_3_	Niacin	Pellagra	Chemical discovery by Weidel 1873; Elvehjem 1937 extraction from liver
B_5_	Pantothenate	Impaired energy production	Essential yeast growth factor by Williams 1933, structure by Williams 1940; Lipmann coenzyme A discover 1946 (Nobel Prize 1953)
B_6_	Pyridoxine	Metabolic disorders	Discovery György 1934, isolation Lepkovsky 1938
B_7_	Biotin	Hair, nail, skin disorders	Bateman 1916, Boas and Parsons 1927, Kögl and Tönnis 1936, among others; consolidated structure by 1940
B_9_	Folate	Anemia	Anemia reversal with yeast by Wills 1941; isolation from spinach leaves [Lat. *sing*. folium] by Mitchel, Snell, William 1941
B_12_	Cobalamins	Anemia	Recognition of pernicious anemia 1847 to 1887, liver concentrate treatment by Whipple, Murphy, and Menot (Nobel Prize 1934), structure by Todd 1955 (Nobel Prize 1957) and via X-ray by Hodgkin (Nobel Prize 1964)
C	Ascorbate	Scurvy	Citrus fruit effects known empirically for long; György, Svirbely, and King late 1920s to mid-1930s, see main text
D	Calciferol	Bone deficiencies	Discovery from cod liver oil by McCollum and David 1914; connection with steroids by Windaus (Nobel Prize 1928); isolation and elucidation by Bourdillon, Rosenheim, King, Callow, and Windaus until mid-1930s
E	Tocopherol/-trienol	Neurological deficiencies	Recognized 1922 and isolated from wheat germ 1936 by McLean Evans; structure by Fernholz 1938
F	Essential fatty acids	General health deficiencies	Discovery and recognition as fats rather than vitamins 1923–1930
G	Now B_2_		
H	Now B_7_		György 1933–1939 (H [German] for Hair (Haar) and Skin (Haut))
I	Not assigned		
J	Choline (or B_2_)	General health deficiencies	Choline isolation by Stricker 1849, elucidated by Baeyer 1957, vitamin J effect proven by Best 1932
K	Menaquinone (K_2_), phylloquinone (K_1_), menadione (K_2_)	Hemorrhages	Recognized by Dam 1929, structure by Doisy 1932 (Nobel Prize 1939)
L	Anthranilic acid	General health deficiencies	Discovery from indigo Fritzsche 1841, structure by Friedländer 1910
M	Now B_9_		Named M after research done in monkeys
N	Alpha-lipoic acid	General health deficiencies	Discovery Snell 1937, elucidation by Reed and Eli Lilly scientists 1950s
O	Carnitine	General health deficiencies	Discovery 1905, structure until 1927, function until 1965, biochemistry Fraenkel since 1950s
P	“Bioflavonoids”	Capillary fragility	Recognition parallel to Vitamin C; see main text
Q-Z	Not assigned		

This strategy also allowed for the application of recent insights from the authors' long-term research on botanical dietary supplements to the dietary role of flavonoids. Our extended body of prior work (see go.uic.edu/botanicalcenter) for an overview and list of 260+ relevant publications. This indicates the importance of several factors contributing to bioactive principles from plants: metabolomic diversity; biosynthetic and metabolic relationships; static and dynamic residual complexity of chemical composition; complexity of biochemical pathways; the multi-layer nature of mechanisms of action; polypharmacology and “synergistic” properties. Collectively, the proven interplay of these factors challenges paradigms that seek to connect, in the most facile manner, single chemical species with well-defined biochemical processes and/or clear-cut biological outcomes.

Accordingly, this study prioritized collecting evidence for the role of VitP as an essential nutritional factor over the question of whether VitP “truly” is a vitamin. The present work followed four hypotheses and addresses three key questions:

(H1) Similar to therapeutic botanical interventions, nutrition acts through multiple, often chemically closely related components, which need to be considered jointly when trying to understand biological effects.
(H2) As the abundance (concentration) and biological significance of plant constituents are uncorrelated, important contributing compounds can be (are frequently?) overlooked when they are minor, difficult to analyze, and/or when the preceding discovery of high-abundance components discourages further or a more in-depth search.
(H3) Common terminology obstructs the quintessential structural differences of congeneric compounds (e.g., flavanonol vs. flavones vs. flavonols vs. chalcones) by using facile common denominators to group chemical entities (“phenol” and “polyphenol”) into inhomogeneous cohorts.
(H4) Combined with the equally common assignment of broad bioactivity terms (“antioxidant”), oversimplifying terminology precludes the ability to establish specific correlations between chemical species and essential biological functions.
(Q1) What is the relationship between flavonoids, VitP, and VitC?
(Q2) Is VitP a single chemical entity or a “complex” of related compounds?
(Q3) What is the role of metabolic, including microbial, transformation in the action of VitP?

In line with these hypotheses and questions, the overarching objective of the study was to compile the available evidence to investigate whether VitP exists, which essential biological role it may fulfill, and which natural compounds can be reasonably associated with these activities. The ultimate goal was to inspire and provide direction for future experimental studies.

## Flavonoids as IMPS and Food Ingredients

Recently, a systematic meta-analysis of world literature on bioactive natural products, encoded in NAPRALERT (2), led to the discovery that certain plant-based metabolites (often considered as “secondary metabolites”) have received massive, but essentially non-productive, attention in the literature. Termed as invalid metabolic panaceas (IMPS), these compounds show bioactivity in virtually all known biological endpoints, frequently due to bioassay “interference,” but despite major research efforts they fail to succeed in their development as drugs (“improbable leads”) or other effective intervention agents such as dietary supplements. IMPS lack the essential characteristics of highly specific leads such as well-defined structure-activity relationships, stability, and other desirable properties. Collectively, IMPS can undermine natural products and nutritional discovery research (1).

However, these findings do not preclude important biological functions for these compounds. In fact, highly abundant molecules may play crucial roles in biological systems—even if their roles are “passive” (i.e., not involving a definable active site, receptor, or analogous target) and not drug-like, but rather function as essential components. While located at the “extreme end” of such a concept, water could still serve as an example. Focusing on phenolic compounds that belong to the group of flavonoids (Supplementary Material 1), the possible existence of un(der)recognized vital biological functions is illustrated in studies of VitP, which was first co-discovered with VitC in the 1930s by Szent-Györgyi.

The close connection between VitP and flavonoids becomes immediately evident when doing literature database searches for “Vitamin P.” Performed with both PubMed and Scopus, they yield in the range of ~800 articles. A manual inspection of all titles and abstracts reveals that almost all these articles published since the mid-1950s use the term “Vitamin P” as a synonym for rutin (vast majority of articles), hesperetin, quercetin, or unidentified flavonoid mixtures (“flavonoids”). The bulk of publications on VitP, however, has been published between 1937 and 1964. Based on these and other insights from database searches that included the refinement of search terms, the present study did not rely as much on database searches, but performed extensive manual back- and forward-tracing of citations in order to generate an as much as possible unbiased and comprehensive collection of reports of experimental outcomes that are directly related to the character and existence of VitP. Tracing evidence back particularly to the earlier VitP literature, including *in vivo* experiments, also provided a means of assessing whether the (over)simplification of “flavonoids” as “Vitamin P” and the idea that rutin could be used as synonymous with VitP were justified, and how these assumptions may have impacted the understanding of VitP in the more recent literature. The authors acknowledge that the manual tracking of literature involves a certain degree of human bias and is limited by the effort that can reasonably be spent. However, the results of database searches led to many, but by far not all, articles cited in the present study. This includes reports that are key to the understanding of VitP, but do not appear in typical database searches, likely due to limitations in their keywords and how the information of these earlier works has been extracted for database purposes.

In order to understand the relationship of flavonoid IMPS and VitP, it is necessary to place the discovery of vitamins and, in particular, VitC into perspective.

## Flavonoids vs. Polyphenols vs. Antioxidants

As workers in different fields have very differing concepts of what constitutes a “polyphenol,” this term has caused much confusion. This has recently been highlighted by a consortium of scientists (3). Because the confounding effect of using “polyphenol” is difficult to avoid even within a well-defined context, the present work avoids the term altogether as it does not add any useful meaning, but actually would introduce more confusion, especially in a broader context. However, one potential explanation and resolution is that the term “polyphenol” was initially intended and used only for poly*meric* (not poly*hydroxylated*) aromatic (“phenolic”) constituents, and that its wider adoption beyond these clear definitions led to a dangerous deviation from its original meaning.

The present focus on flavonoids as a chemical substance class is justified by the available experimental evidence for the existence of VitP. It is important to realize that many flavonoids are not polyphenols as they are not polymeric and only consist of a single flavonoid moiety. Proanthocyanidin researchers typically propose to limit use of “polyphenols” to these polymeric phenylpropanoids and hydrolyzable tannins. In the context of most current uses, the term “polyphenol” is meaningless, should be avoided, and be replaced with the specific type of compound involved in a particular study. This would also bode well on future discussions of DRIs (see below).

Another source of confusion at the interface of chemical structure and bioactivity arises from the fact that virtually all phenols are antioxidants, as they possess unsubstituted (free) phenolic hydroxyl. This suggests that there should not be anything special about flavonoids as antioxidants as an explanation for VitP activity. Interestingly, VitC also is a (powerful) antioxidant, albeit by a very different chemical mechanism. However, with regard to terminology, it is important to emphasize that a present study is neither about “polyphenols” nor about their alleged “antioxidant” effects.

Notably, Health Canada has recently concluded that claims or statements or claims about Oxygen Radical Absorbing Capacity (ORAC) are unacceptable on foods as relationships between ORAC scores and human health effects have not been established. Underlining their strict requirements on antioxidant claims, the authority stated that a specific antioxidant function can be valid when linked to a well-substantiated physiological effect in healthy subjects, as determined by controlled human clinical trials (https://tinyurl.com/48w79wzy).

## Brief History of Vitamins

Early in the twentieth century, the research of several investigators indicated that a family of organic substances found in foods were essential for human life. One of these early investigators, Funk, recognized that small amounts of these substances were essential, and that their absence was responsible for many common diseases such as beriberi, pellagra, and scurvy (4–6). Funk termed these substances vitamins, or “vital amines,” based on the original discovery of thiamin, an amine found to be involved in beriberi. In 1920, Drummond (7) proposed that the term be shortened to “vitamin” as non-amine essential compounds were discovered. He further proposed that thiamine and riboflavin be called Vitamins A and B, respectively, to contrast them from Funk's anti-scurvy factor, which he called Vitamin C (8). Eventually, a series of vitamins was discovered and labeled alphabetically in the order of discovery (Table 1).

This alphabetic listing is primarily historic, because not all vitamins have equally well-defined deficiency diseases, levels of biological activity, and assignments to a single chemical species. For example, the bioactivities of vitamins A and D involve multiple carotenoid and steroidal species, respectively, ranging from nutritionally necessary to actual effector molecules. Furthermore, the B series exemplifies that the vitamin originally designated under one category may turn out to be multiple, in this case even chemically unrelated groups of compounds. Collectively, this means that the historic vitamin designation should be reinterpreted with today's knowledge of complex biochemical systems rather than with the expectation that it is a single compound with a clearly defined biological endpoint.

Although modern definitions differ somewhat, an essential organic chemical compound (or set of related compounds) is called a vitamin when it cannot be synthesized by the organism at all nor in sufficient quantities and, therefore, must be obtained through diet. Thus, the term vitamin is conditional upon the circumstances and particular organism. For example, VitC (ascorbic acid) is a vitamin for humans and guinea pigs, but not for rats and most other animals (9). The well-known Vitamins A, C, D, E, K, and Vitamins of the B complex were among those subsequently discovered in the early part of the twentieth century and later accepted as vitamins (10). In contrast, the proposed VitP was almost forgotten. Putting VitP into proper perspective requires a review of VitC discovery and basic physiology, as follows.

## Vitamin C and Scurvy

By the mid-1700s, scurvy debilitated and killed those whose diet was largely based on meat and starch and devoid of fresh vegetables and fruits. Although the problem was widespread, this was especially difficult for the British navy, as numerous British sailors were afflicted with this disease (10, 11). Attempting to ameliorate this situation, a Scottish physician, James Lind, had observed the curative and preventive powers of citrus fruits and wrote an essay (1757) recommending mandatory consumption of citrus fruits and lemon juice by sailors in the British Navy, eventually leading to their colloquial designation as “limeys.” However, it would take a century for scientists to understand why citrus fruits were so effective against scurvy (10).

Commonly recognized symptoms of scurvy are loss of weight; swollen, soft, spongy, or ulcerated gums; loose carious teeth; hemorrhages; necrosis of the bones; swollen joints; edema; hardening of the skin and often perifollicular or petechial hemorrhages, sometimes bloody conjunctiva and occasionally anemia (12). In the absence of VitC, the development and maintenance of intercellular substances degrades. This involves the collagen of all fibrous tissues and of all non-epithelial cement substances, such as intracellular material of the capillary wall, cartilage, dentin, and bone matrices (12). It has been the prevailing view that scurvy and the deficiency states of VitC are marked by unduly fragile capillaries, despite a considerable body of evidence that there is no direct association of capillary strength and VitC in humans nor in guinea pigs [see (13) and citations therein].

In 1907, two Norwegians, Holst and Fröhlich, reported the existence of a substance that, based on observed biological effects, had the ability to cure the symptoms of scurvy (14). Furthermore, they were able to demonstrate that the absence of this substance produced the symptoms of scurvy in guinea pigs, which are unable to synthesize the substance endogenously. This work was largely ignored because, at that time it was generally accepted that only lipids, proteins, and carbohydrates were needed for growth and development of animals.

In approximately 1924, the Hungarian scientist Szent-Györgyi began to study animal, vegetable, and synthetic oxidizing systems (15). By 1928, he had isolated and accumulated about 30 grams of a strongly reducing substance, which he called hexuronic acid, from adrenal tissue, citrus species, and cabbage. Further, Szent-Györgyi also provided a sample of hexuronic acid to Haworth, an eminent chemist who, in turn, passed the sample to colleagues who determined the structure (16). Interestingly, in his early studies, Szent-Györgyi did not carry out bioassays to establish that his substance was the antiscorbutic compound, but provided a sample to another investigator, Zilva, who, in 1932, declared that Szent-Györgyi's hexuronic acid was not VitC (17–20).

Perhaps slightly after Szent-Györgyi had begun his studies on hexuronic acid in 1928, King at Pittsburgh began complementary studies. King's contribution involved the isolation of VitC from lemon juice in 1931–1932 and study of its antiscorbutic activity in guinea pigs (21, 22). The research groups of King and Szent-Györgyi connected when a Hungarian-American, Svirbely, who worked with King at the University of Pittsburgh until 1931, returned to Hungary and worked with Szent-Györgyi. By early 1932, Svirbely had established that hexuronic acid was the antiscorbutic factor identical to VitC (16, 23–25). Within 2 weeks of each other in the spring of 1932, first Waugh and King (26), King and Waugh (27), and then Svirbely and Szent-Györgyi (16) published articles declaring that VitC and hexuronic acid were the same compound, thus VitC was subsequently named ascorbic acid (28, 29). Later work by Szent-Györgyi and his collaborators, with the diet of Sherman et al. (30) for induction of scurvy in guinea pigs, was the first to demonstrate VitC avitaminosis (16, 24) and finally explained its link with the treatment and prevention of scurvy (11).

Szent-Györgyi was awarded a Nobel Prize in Medicine in 1937 for his work with regard to VitC. However, controversy remains over whether both Szent-Györgyi and King deserved equal credit for the discovery of VitC. Szent-Györgyi's further accomplishments included the discovery of the role of adenosine triphosphate and actin-myosin, elucidation of many phases of the Krebs cycle, and studies on the influence of free radicals in tumor formation (31, 32). Haworth received the 1937 Nobel Prize in Chemistry for his investigations on the chemistry of carbohydrates and VitC.

## Vitamin P Emerged From Vitamin C Research

Many biochemical processes involve VitC and its oxidized counterpart, dehydroascorbate, as cofactors: hydroxylase metabolism; (nor)adrenaline biosynthesis in the adrenal cortex; formation of dopamine and noradrenaline (syn. norepinephrine) in adrenergic synapses; and metabolism of aromatic amino acids (Supplementary Material 5). The most characterized function of VitC involves collagen biosynthesis, where it takes part in the post-translational hydroxylation of prolyl and lysyl residues in unfolded procollagen chains, which is essential for folding collagen into triple helices prior to collagen secretion by fibroblasts. Hydroxyproline residues contribute to the stiffness of the collagen triple helix, and hydroxylysine residues hydrogen bond with carbohydrates and form intramolecular cross-links that give structural integrity to the collagen mass. The under-hydroxylation of procollagen, which is degraded, appears to be a major factor in the pathophysiology of scurvy. VitC deficient subjects usually show reduced urinary excretion of hydroxyproline (33).

From the collagen pathophysiology perspective, the VitC story is quite satisfying. An avitaminosis condition could be treated by a single biologically active compound, designated as VitC, and chemically identified as ascorbic acid. Structure activity relationships have been established (Supplementary Material 2) and specific biological targets identified. However, the VitP story emerges from the VitC narrative. Although Szent-Györgyi was awarded the Nobel Prize for the discovery of VitC, according to some, he was never fully satisfied, because his experiments with VitC showed something was “missing” (22, 29). This is where VitP enters the picture.

Early VitC studies were performed with natural product extracts, which contained VitC along with other compounds (impurities) that accounted for the residual complexity, i.e., minor chemical species in nature-derived agents that impact biological test systems and bioactivity profiles of the assay material (34). VitC became the focus of these studies as it was the major component of the extracts. Moreover, the role of putative impurities was obscured by the apparent success of VitC to reverse most, but not all, biological effects of a scorbutic diet in guinea pigs and humans.

## Early Evidence for Vitamin P as a Flavonoid

Although the structure of VitC was still unknown in the 1920s, several investigators at the time provided evidence that VitC interacted with a second factor of unknown composition (35–38). Based on these reports and his own experiments, with isolated and characterized VitC, Szent-Györgyi also believed that there was an additional chemical factor that played a significant role in curing scurvy (22, 29). He felt that this additional substance was of similar importance and related activity with VitC. In the dietary absence of both VitC and this unknown entity, the symptoms of scurvy prevailed and concealed symptoms of the deficiency of the second substance (39). Along with other investigators, he found that in certain pathological conditions characterized by an increased permeability or fragility of the capillary wall, highly purified or synthetic VitC was ineffective for reducing the permeability, whereas the condition was readily cured by administration of extracts of Hungarian red pepper (called “Vitapric”) or lemon juice. These extracts were effective in cases of decreased resistance of the capillary wall toward whole blood (vascular type of hemorrhagic purpura), as well as in cases where the capillary wall showed increased permeability toward plasma protein only, such as observed in various septic conditions. As little as 40 mg of this active fraction given daily intravenously to a human restored normal capillary resistance in 2 weeks. Spontaneous bleeding ceased, the capillary walls lost their fragility toward pressure differences, and no more plasma protein left the vascular system on increased venous pressure (39).

In a second publication, Szent-Györgyi et al. demonstrated that when guinea pigs were fed a diet that induced scurvy, those that also received 1 mg daily of the crystalline flavonoid fraction of lemon juice (called citrin), survived much longer than those that did not. Those animals not receiving citrin died in 28 days, whereas those with citrin lived 44 days on average. Both groups showed typical symptoms of scurvy, but the group that did not receive citrin had major increases in hemorrhages of several types in comparison to group that received citrin (40, 41). The authors argued that these results indicated that experimental scurvy, as it is commonly known, is a deficiency disease caused by the combined lack of VitC and other components of lemon extract. Accordingly, they proposed the name “Vitamin P” (VitP) for the substance responsible for the action on (vascular) permeability. The decision to call this material a “vitamin” later proved to be a source of controversy (31). Experimental difficulties in preparing a strictly VitP deficient diet and, thereby, establishing a hard biochemical link to a deficiency syndrome are one reason why the scientific community has found it difficult to embrace VitP as a vitamin. The intertwinement with VitC further obscures the potential link between deficiency and disease. However, as discussed below, evidence for VitP as an essential nutritional factor should take precedence over the formal question of its designation as a “true” vitamin vs., e.g., being a micronutrient.

Zacho investigated the impact of citrin (see Supplementary Material 3 for a comprehensive description) in capillary resistance in guinea-pigs (*n* = 36) fed with various diets using the sucking-cup method adopted from the human clinical use to the animal model (42). His findings confirmed that citrin was effective in restoring normal capillary resistance from scorbutogenic dietary conditions. Unfortunately, several workers, notably Zilva (19, 43), were not able to duplicate the effects of citrin and its interactions with VitC as claimed by Moll (44), Lotze (45), and Parrot and Sevestre (46). Even Szent-Györgyi was not always able to duplicate his own results (47, 48). However, in other cases, the initial observations of activity of citrin and other flavonoid fractions could be repeated (13). Although the difficulty to repeat the original work may have been for a variety of reasons, one major problem was that the preparations used by Szent-Györgyi et al. were not consistent in composition (46, 47, 49–51). From today's perspective, these reported reproducibility issues could be interpreted as a form of (chemical) residual complexity. Researchers attempted to isolate the active component of citrin. Upon fractionation, the active fraction from lemon juice was found to consist primarily of flavonoid glycosides. The experimental description of the production of citrin, the phytochemical methods available at the time, and the incomplete compound purification (without crystallization to constant m.p.) most likely yielded materials with variable chemical composition.

Despite the fact that Armentano et al. (52) found that use of lemon juice and preparations of paprika rich in VitC produced favorable results in certain patients with bleeding diseases, namely vascular purpura and protein permeability of the capillaries, the same results were not observed with purified VitC. From these studies, it appeared that purified and synthetic VitC were less effective than unpurified VitC from natural sources for treating thrombocytopenic purpura. These observations reinforced the notion that bioactive minor compounds, attributed to residual complexity, were present in the VitC-rich extracts. Lavollay demonstrated that injection of pure, synthetic VitC caused elevation of capillary resistance in non-scorbutic guinea pigs (2 mg per 400 g animal), whereas injection of the flavanone, epicatechin (1 μg), was 10,000–20,000 times more effective than VitC. Others also have not detected any increase in capillary resistance following administration of VitC alone to apparently healthy persons on inadequate diets or to persons with scurvy (45, 49, 52, 53). Interestingly, a very recent meta-analysis of 17 qualifying clinical trials has associated the flavonoid quercetin with lowered systolic blood pressure as a clinical endpoint, in the absence of lipid and glucose metabolic effects (40).

Even today, residual complexity plagues programs aimed at natural product discovery. The ambiguities in the reported interaction of flavonoid preparations such as citrin and VitC foreshadowed the ongoing difficulties that have been experienced with performing biological assays on nature-derived materials. The intricacies of purity and the chemical integrity of assay materials has recently been recognized as a persistent problem in biological assays (54).

## Expanding the Repertoire of Vitamin P Candidates

**Figure 1:**
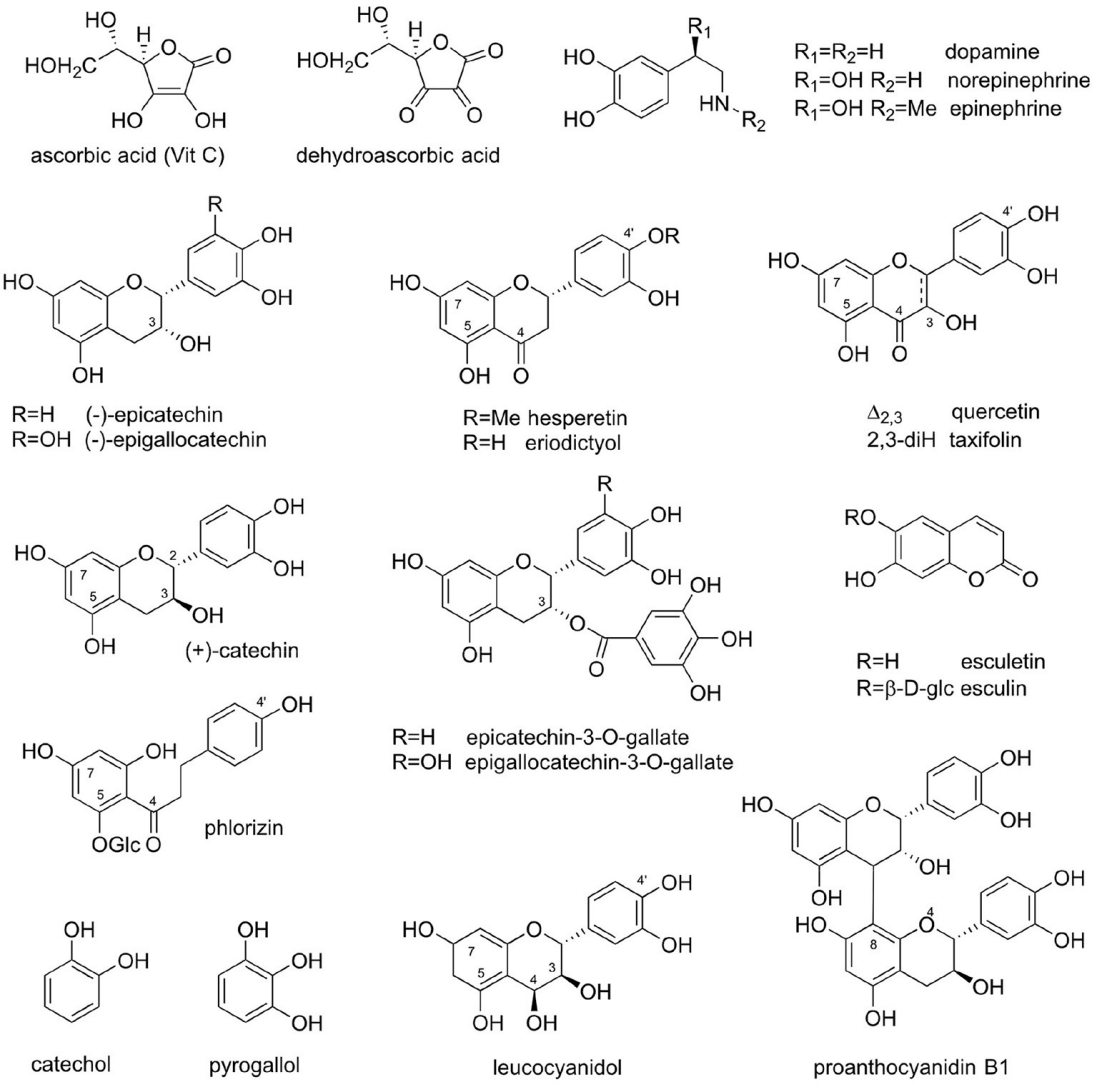
Natural products featured in the Vitamin P story: Vitamin C (ascorbic acid); dehydroascorbate; dopamine, noradrenaline (norepinephrine), and adrenaline (epinephrine); hesperetin and eriodictyol; (–)-epicatechin and (–)-epigallocatechin; quercetin and taxifolin; (+)-catechin; (–)-epicatechin-3-*O*-gallate and (–)-epigallocatechin-3-*O*-gallate (EGCG); esculetin and esculin; phlorizin; catechol; pyrogallol; leucocyanidol; and proanthocyanidin B1 [epicatechin-(4β → 8)-catechin].

With the VitP hypothesis in hand, a search was undertaken to identify a chemical principle that enhanced the activity of VitC for treating the cause and symptoms of scurvy. A major problem in the search for a specific compound that could be identified as VitP proved to be that a series of structurally diverse flavonoids from other sources also produced similar results. For example, the glucosides of the flavonoid aglycones, hesperetin, and eriodictyol, were both active. In comparison, the flavonoid fractions of *Citrus* extracts contained heterosides of quercetin. In some cases, comparative activities were measured, for example, citrus fruit concentrates were reported to have 20 times the activity of hesperidin (49, 51). In his publications, Szent-Györgyi concluded that VitP should be a flavanone with exceptional properties owing to its activity vis-à-vis oxidative agents (47). However, Javillier and Lavollay noted that the activity was not associated with specific flavonoid structures, but also with many flavonoids including flavonols, flavanones, and their glycosides, as well as catechins and their oligomeric proanthocyanidins [Figure 1; (50)].

Catechins from cutch (*Senegalia catechu* [syn. *Acacia catechu*], catechu) proved to be a source of VitP-like activity. Although Lavollay et al. found the original crystalline (+)-catechin from cutch to be inactive (55, 56), the mother liquor was quite active. Notably, (–)-epicatechin, from the mother liquors was 500–1,000 times more active than the flavonoid fraction from citrus extracts (55–57). This example of “dynamic” residual complexity (54) plausibly had resulted from an epimerization event that occurred during recrystallization of a compound isolated by bioassay guided fractionation. Based on LC/MS and LC/MS/MS analyses, it is now known that the major monomeric catechins of cutch are (+)-catechin, (–)-epicatechin, epicatechin-3-*O*-gallate, and epigallocatechin-3-*O*-gallate (58). Extracts of gambier, *Uncaria gambir* (Rubiaceae), a source of the catechin oligomers called proanthocyanidins, were also found to be active. Gambier extracts contain primarily (+)-catechin and only small amounts of (–)-epicatechin (59).

In subsequent studies, the French investigators found that coumarin and the coumarin derivatives esculin and esculetin also were quite active (60). A flavonoid dihydrochalcone, phloridzin, even showed activity in microgram quantities (50). Coumarins are widespread in nature, however, phloridzin is best known as an apple skin component and is only occasionally found in other plants.

In 1948, Masquelier founded a company that prepared an extract of the bark from French maritime pine, *Pinus pinaster* Aiton subsp. *atlantica* Villar, called Pycnogenol^®^, that had VitC-like properties [Supplementary Material 4; (61)]. Chemical identification studies showed that this extract is primarily composed of proanthocyanidins that are biopolymers of (+)-catechin and epicatechin with two or more flavonoid subunits. The extract also contained monomeric (+)-catechin and epicatechin.

Lavollay and Sevestre demonstrated that Bordeaux wine, from which the ethanol was removed, possessed Vitamin P activity when injected into guinea pigs and humans (62). Masquelier et al. later discovered the presence of oligomeric proanthocyanidins in red wines (63, 64). About 1970, it was discovered that grape seeds were also rich in oligomeric proanthocyanidins. Early research on the effects of grape seed extracts on the permeability of vascular capillaries was designed to unravel the underlying cellular and molecular mechanisms of action of oligomeric proanthocyanidins and other flavonoids (65).

During the 1970's, Masquelier began producing a grape seed extract for medicinal uses as an alternative to the pine-derived Pycnogenol^®^. Grape seeds contain a similar, but not identical complement of the oligomeric proanthocyanidins (OPCs; syn OPACs) to pine bark. Masquelier's product, called “MASQUELIER's^®^ Original OPCs,” was extracted from the seeds of *Vitis vinifera* L. This product contained catechin, epicatechin, and OP(A)Cs (dimers to pentamers) (66). Detailed analyses have established that the product is standardized to contain about 85% (w/w) flavonoids, of which 50–60% (w/w) are monomeric and dimeric catechins. The same product was evidently devoid of OP(A)Cs (65, 67).

*In vitro* studies indicate that Pycnogenol^®^ constituents demonstrate a high percentage of binding to collagen fibers, promote synthesis of collagen and elastin, and inhibit their proteolytic degradation (68). Masquelier's OP(A)Cs have been demonstrated to bind collagen and elastin in blood vessel walls, promote collagen synthesis and polymerization, and inhibit degradation of collagen and elastin in a study conducted in guinea pigs (69). The OP(A)Cs of grape seed show strong collagen protection. By binding to collagen, they also may offer protection of elastin and collagen in vascular tissues from degradation by elastase and collagenase, respectively (65).

Flavonoids such as (-)-epicatechin and dimeric procyanidins in human diets can be absorbed and reach the bloodstream and other organs (67, 70, 71). OP(A)Cs consisting of larger numbers of monomeric units are poorly absorbed and mostly pass through the digestive system unaltered. In this process, a portion of certain OP(A)Cs bind to membranes of the gastrointestinal tract. These substances have a key role in stabilizing membranes by preventing their disruption via chemical and biological agents, and regulating membrane-associated events. In many instances, OP(A)Cs appear not to bind directly to degradative enzymes, but to bind to matrix macromolecules and prevent their degradation by various factors such as temperature, oxidative stress, inflammation, and proteinases (67, 70–74). The original lemon and paprika extracts studied by Szent-Györgyi and others are rich in VitC and a variety of flavanones, but essentially lack catechin and proanthocyanidins (75–77).

Fibrillar collagen is a strong and viscoelastic biomaterial arranged into highly organized hierarchical structures. Type 1 collagen is the most abundant of all collagen types and is defined as an interwoven coiled trimer, containing repeated sequences of proline and hydroxyproline (78). The interaction of OP(A)Cs and collagen is believed to be stabilized by hydrogen bonding between the protein amide carbonyl and hydrophobic bonds. The relatively great stability of OP(A)C-protein complexes suggests structural specificity. Indeed, two new trimeric and tetrameric A-type OP(A)Cs capable of strengthening the micromolecular backbone of teeth via intermolecular and intermicrofibrillar cross-linking have been reported (79). The applications in dentistry include disease prevention (caries) and partial tooth repair.

In conclusion, while astute experimental observations concerning the residual complexity of early VitC preparations from natural sources led to the VitP hypothesis, the residual complexity of VitP formulations (also prepared from natural extracts) led to considerable confusion in identifying a “lead compound” in the search for Vitamin P. It has not been possible to attribute VitP activity to a specific food, preparation, or compound. This problem, coupled with the difficulty in attributing a deficiency disease that could clearly be linked to Szent-Györgyi's lemon extracts or to VitP might have led scientists to abandon VitP as a useful hypothesis. Nonetheless, despite the difficulties in understanding the relation of Vitamins C and P, two things have been established: (i) the existence of a distinct biological effect of VitP, i.e., the influence of a series of naturally occurring flavonoids and coumarins (Figure 1 and Supplementary Material 1) on vascular permeability; and (ii) the influence of these natural products on the anti-scurvy VitC effect (45, 49, 65).

## Evidence for Vitamin P Avitaminosis

One of the early objections to consideration of VitP as a true vitamin lay in the difficulty of establishing disease symptoms that were related to the identified series of compounds. This shortcoming was addressed by later studies of Casley-Smith et al. who worked mostly with rats, which have the ability to synthesize VitC endogenously. When fed a diet lacking flavonoids, the rats exhibited definite structural alterations in blood capillaries and tissues. A diet of this type, for the time period employed, produced considerable increases in capillary fragility. These fine structural alterations were quite different from those reported for VitC avitaminosis and implied a different deficiency (80, 81). Other work demonstrated that lack of flavonoids gives rise to cerebral edema due to the increased permeability of the blood-brain barrier (82). In skin, a flavonoid-deficient diet greatly increased capillary fragility, which was reversed by the addition of flavonoids to the diet (80).

Medicinal preparations involving a semisynthetic flavonoid compound, *O*-(β-hydroxyethyl)-rutoside, were developed about 1960 (83). The corresponding preparation, Venoruton^®^, has been widely used in Germany and Switzerland for the treatment of edema and other vascular disorders. Oral administration of Venoruton^®^ increased conjunctival capillary resistance in rabbits. This preparation has few negative side effects and, importantly, is soluble in water, whereas rutin and other flavonoids with similar activity have limited water solubility.

Studies with guinea pigs and humans implied that natural flavonoids and coumarins acted as vitamins in those animals, and that VitC and VitP deficiency states are quite distinct. The structural effects of VitC deficiency in both guinea pigs and humans have been studied by a number of workers who noted a significant reduction in the amount of collagen, with swelling of the fibroblasts and endoplasmic reticulum. The basement membranes of the blood capillaries (and associated lymphatics) were often very tenuous and disrupted, although they sometimes appeared thicker (80). In contrast, in VitP avitaminosis in rats, the basic lesion consisted of the opening of some blood capillary endothelial intercellular junctions. Unlike in VitC avitaminosis, the endothelial cells were intact, without a pale, grossly swollen cytoplasm (80). These effects were largely prevented by feeding coumarins as well as flavonoids such as troxerutin (80, 83, 84).

The studies by Casley-Smith implied that the natural flavonoids and coumarins were vitamins. VitC and VitP deficiency states are sufficiently distinct to justify the assignment of VitP as the underlying factor. It seems probable that some of the changes in VitC deficiency observed in many studies were due to a concomitant VitP deficiency. Whereas both conditions had many open endothelial junctions and somewhat altered basement membranes, all the gross distortions of the endothelial cells observed in VitP avitaminosis were considerably lessened by flavonoids and coumarins.

## Early Ideas on the Mechanism of Action of Vitamin P

Probably because adrenal glands were an original source of VitC, and due to the fact that adrenaline was a major factor in capillary blood flow, much attention was focused on adrenalin as a link to VitP activity (49, 62, 85). *In vitro* experiments showed that adrenaline is readily oxidized by catechol oxidase, the cytochrome system, amine oxidases, and peroxidases (86). Javillier and Lavollay supposed that VitP slowed down oxidation and, thereby, the resulting inactivation of adrenaline (50, 87–89); adrenaline can also be inactivated by non-oxidative enzymes, such as catechol-*O*-methyltransferase. The ability of many different types of flavonoids and coumarins to affect the persistence of adrenaline was evaluated in a subsequent extensive study (85).

It is likely adrenaline is involved in the action of VitP: it binds to α-1 receptors that are involved with vasoconstriction, smooth muscle contraction of the bladder neck, and glycogenesis. Adrenaline also binds to α-2 receptors, which are involved with vein constriction, central attenuation of the sympathetic nervous system, inhibition of insulin release, and relaxation of the intestine. Other interactions include β-1 receptors that provide positive inotropic and positive chronotropic signals to the heart, lipolysis, and renin release, as well as β-2 receptors, providing bronchodilation, vasodilation, gluconeogenesis, relaxation of the uterus, and relaxation of the intestine. The ubiquitin receptor (vascular dopamine) DA1 is involved with vasodilation, whereas the DA2 receptor prevents presynaptic noradrenaline release [http://www.urology-textbook.com/adrenal-glands-catecholamins.html; (90)]. The plasma half-life of adrenaline is ~20 s. Inactivation depends mainly on monoamine oxidase (MAO) and catechol-*O*-methyltransferase (COMT) availability.

Early experiments with flavonoids examined their role in the metabolism of adrenaline. Injection of quercitroside inhibited decomposition of adrenaline in dogs and cats. In addition, quercitroside extended the physiological action of adrenaline in cats (46). In guinea pigs that were scorbutic, a number of other substances were shown to inhibit oxidation of adrenaline *in vitro*, however, most of them, e.g., catechol and pyrogallol, were not substances that would normally be found in the diet or in animals (56, 57).

Alternatively, other investigators concluded that the activity of VitP consisted of inhibiting the oxidation of VitC. In 1947, Masquelier isolated a flavan-3,4-diol, leucocyanidin (syn. leucocyanidol), from the seed coats of peanuts (91–93). He found that extracts containing flavan-3,4-diols could protect VitC from oxidation *in vitro*. He also demonstrated that rutin, esculin, and proanthocyanidin B1 all inhibited *in vitro* oxidation of VitC mediated by Cu^2+^ ions and ascorbic acid oxidase. In subsequent work, Masquelier referred to “leucocyanidol” as “OPCs” (oligomeric proanthocyanidins) although in his 1951 papers, he refers to the tested substance as a monomer (91–93). Later, Bate-Smith and Ribéreau-Gayon confirmed that the original “leucocyanidol” isolated was indeed a flavan-3,4-diol (94). Overall, Masquelier examined the health benefits of probable mixtures of these compounds and based on his subsequent studies, developed a vasculo-protective medicine in 1950. However, the (somewhat surprising) limited availability of peanut skins necessitated examination of other plants sources of active antioxidant substances (29).

At first sight, the role of VitP as an “antioxidant” in biological systems might appear an attractive explanation for many aspects of the biological role of flavonoids, because it can easily be rationalized why so many diverse flavonoids and related phenylpropanoids display VitP activity. However, “antioxidant” bioactivity does not adequately explain the apparent link between VitC and VitP activity, especially as VitC could, presumably, act as its own antioxidant. Flavonoid compounds such as flavan-3-ols, flavan-3,4-diols, and oligomeric proanthocyanidins (including procyanidin B1) have radical scavenging activity and, therefore, have been proposed to serve as “antioxidants.” It is now known that vascular function is strongly influenced by oxidative stress and that diminishing oxidative stress also reduces inflammatory stress (65). However, flavonoids and related compounds, as well as extracts containing these compounds, may be involved in a multitude of biological functions and may have multiple (pleiotropic) effects that during evolution have not been selected for a strong effect on a single well-defined target (65, 67), but rather the opposite.

## Plausibility of Flavonoids as Bioactive Compounds

The NAPRALERT Database (2) was consulted to investigate the plausibility of flavonoids as bioactive compounds. Plant natural products from 421 families, 3,714 genera, and 16,011 species are represented in NAPRALERT. A much smaller number of plants have been examined for biological activity. A NAPRALERT search revealed that ~50,000 biological experiments have been published on flavonoids. About half of these are based on commercially available materials. The other half included flavonoids from about 1,700 species of living organisms, primarily plants. Almost 7,000 compound names associated with these experiments are represented. The qualitative activity, i.e., overall active to inactive activity, reported for these compounds is 2:1 (3:1 *in vivo*) which means that 66-75% of flavonoids have been reported as being bioactive in their respective assays.

Flavonoids also have been reported to have bioactivity in a very broad variety of bioassays. They undoubtedly interact with many different systems in mammals. These pleiotropic interactions may be detrimental or beneficial. Note that flavonoids and related phenolics often interfere with bioassays and, therefore, are described as pan-assay interference compounds (PAINS) (95). PAINS interfere with *in vitro* assays through various mechanisms including fluorescence, redox, or through generalized binding to enzymes. As a result, flavonoids represent 10 of the 22 natural products that have been identified as most likely being natural product invalid metabolic panaceas (IMPS) (1).

## Are Vitamin C and Vitamin P Mutual Co-Factors?

One early experimental approach to explore this question used guinea pigs, which like humans, lack the ability to synthesize VitC. They were divided into five groups. A control group was given VitC, whereas other groups received no or sub-optimal levels of VitC, but were given various levels of OP(A)Cs. Even though deprived of VitC, those that received an appropriate amount of OP(A)Cs survived as long as those that got adequate amounts of VitC. Lotze concluded that VitC and OP(A)Cs have a marked “synergy” because flavonoid co-administration of OP(A)Cs and VitC made it possible to decrease the dosage of VitC (22, 45, 53, 96). Pleiotropic effects (multiple biological effects from the same phytoconstituent) may also explain bioactivity that cannot be rationalized by classical reductionist models (67).

## Does Metabolic Transformation Contribute to Flavonoid Bioactivity?

When considering bioactivity research that is dominated by live animal assays, it is necessary to consider the contribution of the dynamic complexity of metabolites to the observed activity. The stability and metabolism of catechins and other flavonoids under digestive conditions is relatively poorly understood. However, several common food additives including VitC, milk, and citrus juice enhance the stability of epicatechin, epigallocatechin, epigallocatechin gallate, and epicatechin gallate, when incorporated into tea beverages. From *in vitro* digestion studies, without additives, <10% of epigallocatechin gallate and epicatechin gallate were recovered. Addition of VitC increased recovery to 54 and 74%, respectively, while epicatechin recovery was 82%. Of all substances tested, juice preparations promoted stability best. Epicatechin recovery was then between 86 and 95%. Of the juices tested, lemon juice was most effective. Catechins are most stable in aqueous solutions at about pH 4 (97, 98).

VitC and VitP may play a joint role in the biological activity of VitC (99, 100). VitC reacts with *p*-hydroxybenzyl alcohol to produce two epimeric forms 2-*C*-(*para*-hydroxybenzyl)-3-keto-hexulosonic acid (99). These authors also indicated that VitC can react with leucoanthocyanidins (flavan-3,4-diols) and proanthocyanidins (such as proanthocyanidin B1) to produce adducts. Both epi(gallo)catechin and epi(gallo)catechin-3-*O*-gallate are very similar with regard to structure and reactivity (100).

Metabol(om)ic relationships including xenobiotic conversions, which exist between the flavonoid structures and structurally distinct derivatives and/or reactive species may be the actual carriers of the VitP effect. Recently evolving studies provide compelling evidence for gut microbiota and microbiome playing a key role in intestinal flavonoid metabolism. Recent reviews and studies [see (101–103) and references therein] have already summarized the impact of such processes on flavonoid bioavailability, the deglycosylation and formation of metabolites that unfold the actual effects at the site of action, the inter-individual differences of health outcomes, and connections with cardiovascular health.

In addition to the property of flavonoids to form a network of readily interconverting congeners that primarily differ in the degree of (un)saturation and oxidation of the pyran/C-ring, flavonoids yield a common set of very small molecule degradation products when exposed to gut microbes. Among these microbial metabolites are hydroxybenzoic acids (HBAs), including salicylates, and other small phenolic acids (104). Considering the relatively high abundance of many flavonoids, HBAs can be produced in physiologically relevant amounts. Existing evidence regarding the catabolism of the flavan-3-ol subgroup has been compiled and discussed in recent reviews (105, 106).

## Can We Shed Light on Historical Reports?

Unambiguous data show that the activity of Vitamins C and P are related. In a number of studies, guinea pigs on a scorbutogenic diet developed symptoms of scurvy and the animals also developed reduced capillary resistance. This latter effect was reversed by addition of citrin, hesperidin, or other flavonoids to the diet. Interpretation of data from different methods for determination of capillary resistance and lack of experimental details contributed to variation in the results of many of the studies reviewed by Scarborough and Bacharach (49).

Other problems result from the composition of scorbutic diets used to study the role of VitC and VitP in guinea pigs and rats. Many early studies, including those of both King and Szent-Györgyi, were based on a scorbutic diet that consisted mainly of freshly ground oats (*Avena sativa*), dried milk powder, salt, and butter fat. Notably, oats contain (–)-epicatechin, a compound with pronounced VitP-like activity (107). The presence or absence of (–)-epicatechin and related flavanols in the diets employed, opens questions about the interpretation of the results of many of these early studies, and/or the role of (–)-epicatechin in VitP activity.

Nonetheless, we concur that VitP activity is real. At the same time, the strict definition of a vitamin as a single chemical entity (SCE), the absence of which generates a disease (avitaminosis), does not apply because of the complex composition and interrelated nature of the compounds that can serve as VitP. In this respect, VitP resembles a complex similar to Vitamin B. While the definition of Vitamins P1, P2, P3, etc. is premature, they differ from the B-series by being structurally related.

Much of the vast reported evidence suffers from being chemically inconclusive, due to a lack of rigor in the chemical characterization of the intervention materials. While this, in part, reflects the progress in chemical and structural analysis made since performing the biological assays that characterized the vitamins, other potential explanations for the gap in our current understanding of VitP may lie in the following shortcomings: (a) a general trend toward bioassays that are driven by reductionist hypotheses (single agent, single target) vs. consideration of pleiotropic activity/targets (multiple biological effects originating from the same phytoconstituent); (b) the general lack of consideration of metabolic activation, in particular the conversion of flavonoids with poor PK properties into metabolites that can be absorbed; (c) a tendency to ignore all forms of residual complexity, particularly the impurity of the natural products used in bioassays (static RC), as well as stability/conversion during bioassay and/or chemical processing (e.g., heating during recrystallization, triggering epimerization and other chemical reactions).

## Is Vitamin P a Single Chemical Entity? Is it a Vitamin?

Considering the evidence available to date, VitP does not exist as a single chemical entity, but consists of a group of analogous, and often congeneric, compounds. The search for VitP will unlikely lead to a single isolated compound. This challenges existing paradigms built on more simplistic ligand/target models and indicates that VitP function involves higher complexity on both sides of biological action, i.e., the chemistry of the agents and the biochemistry of the biological targets and networks. As mentioned above, a number of flavonoids and flavonoid glycosides have been considered to be VitP. However, these compounds mostly have limited activity in comparison to certain coumarins (e.g. esculetin; Figure 1), as well as the flavonoids phloridzin and epicatechin. Epicatechin and oligomeric catechins (such as procyanidin B1) are widely distributed among plants and are found in many food plants.

Whether the catechins and/or OP(A)Cs constitute a vitamin (complex), related or unrelated to VitC, is, in part, a question of definitions. Notably, it has not been demonstrated unambiguously that VitC *alone* is adequate for resolving the symptoms of scurvy (49). In the opinion of Masquelier, flavan-3-ol oligomers (i.e., catechol oligomers or oligomeric proanthocyanidins [OP(A)Cs]) are the only flavonoids that have a justified claim to VitP activity (22). Furthermore, there is significant evidence that coumarin derivatives, which share a biogenetic and ADMET relationship with flavonoids, have VitP activity and also interact in a powerful manner with VitC (45, 53). Notably, while representing constituents present in certain dietary plants, coumarins themselves are excluded from becoming dietary supplements due to their approved drug status.

Although VitP has not been widely accepted as a vitamin by the scientific community, numerous commercial dietary supplements are currently sold as “VitP,” sometimes using synonymous terms such as “bioflavonoids.” The variety of chemical structures associated with commercial products sold with the label “VitP” is as diverse as all the structures shown in this article (Figure 1), with the exception of the coumarins, and always has a focus on flavonoids.

As detailed above, the designation of VitP as a vitamin has historical roots and stems from the era of vitamin discovery (ca. 1918–1948; Table 1), when nutrients present at relatively low concentrations in certain foods were recognized as causing deficiency symptoms or diseases and as being essential, and were labeled in a more or less organized alphabetical order of discovery. Compared to the well-established vitamins, VitP is chemically elusive yet biologically relatively well-documented. Reflecting the overall complexity of describing it chemically and biologically, its status as a vitamin remains hypothetical. Its “metabolic network chemistry” and interdependence on other “co-factors,” particularly its established relationship with Vitamin C, may eventually move its label from vitamin to the broader micronutrient category. However, in the view of the authors, the elusive and partially hypothetical nature as well as its to-be-resolved classification tends to hide the importance of VitP and certain “bioflavonoids” as an essential factor of human health.

## What About Dietary Reference Intakes of Flavonoids?

Based on all available/reviewed data, it is currently not feasible to define Dietary Reference Intakes (DRI) for VitP. Review of recent references does not clarify how the DRI of flavonoid substances can be accomplished. Several recent reports (108–111) outline how DRIs can be approached for certain compounds. Accordingly, DRIs are potentially feasible for the carotenoid lutein (not to be confused with the flavonoid, luteolin), but it is impossible to carry out the required steps for flavonoids. Although a single compound may ultimately be shown to provide most VitP activity, a number of congeners may be involved in its interaction with VitC. Collectively, much additional information will be required before determination of a VitP DRI is possible.

## Potential Directions for Essential Flavonoid Research

Flavonoids are apparently closely related, yet still form a rather heterogeneous group of metabolically interchangeable essential nutrients with poorly understood biological profiles. The historic and ubiquitous nature of flavonoids in the human diet suggests that they may have become essential dietary nutrients at some point in the evolution of the human species. In fact, it is difficult to devise a diet that is completely deficient in these compounds as flavonoids cover a wide range of chromatographic polarity. Moreover, the highly generic nature of flavonoids as bioactive compounds suggests that no single compound emerged as playing a decisive role. This seems to indicate that a more generalized activity might be at work, which is strikingly well-aligned with the *Screening Hypothesis* by Firn and Jones (112) and Jones and Firn (113).

At the same time, this does not rule out the possibility of specific biological targets, which remain to be determined. In fact, there may be multiple specific biological targets that are impacted by various chemical members of the VitP family (65, 67). The relevant literature associates VitP activity with 12 flavonoids (Figure 1 and Table 2), which belong to various subclasses (flavanones, flavan-3-ols, flavans, flavanonols, chalcones, flavan-3,4-diols, and proanthocyanidins), each of which has a large number of closely related and relatively widely occurring congeners. Thus, the total number of structural and metabolically related flavonoids that contribute to the overall VitP activity could be substantial. At the same time, some members of this “flavonoid network” likely have more pronounced VitP bioactivities than others. In the understanding of the authors, based on the evidence summarized above, the flavan-3-ols including the proanthocyanidins could potentially be such an important subclass.

**Table 2:** Description of the compounds shown in Figure 1 with an emphasis on their role in the vitamin P story.

**Compound**	**Class**	**Origin**	**Known or proposed interactions**
Ascorbic acid	γ-lactone	Not biosynthesized in humans, but prevalent in many plant and animal species	VitC and VitP work together
Dehydro-ascorbic acid	γ-lactone	Not biosynthesized in humans, but prevalent in many plant and animal species	
Dopamine	Catechol Phenylethylamine	Mammalian neurotransmitter	VitP slows down oxidation of dopamine *in vivo*
Norepinephrine	Catechol Phenylethylamine	Mammalian neurotransmitter	VitP slows down oxidation of norepinephrine *in vivo*
Epinephrine	Catechol Phenylethylamine	Mammalian neurotransmitter	VitP slows down oxidation of epinephrine *in vivo*
Epigallocatechin	Flavan-3-ol Pyrogallol	Plant-derived natural product	Likely present in plant extracts that show VitP activity
Epicatechin	Flavan-3-ol Catechol	Plant-derived natural product	Present in plant extracts that show VitP activity. Shows VitP activity as purified compound
Hesperetin	Flavanone	Plant-derived natural product	Glucosides present in plant extracts that show VitP activity
Eriodictyol	Flavanone Catechol	Plant-derived natural product	Glucosides present in plant extracts that show VitP activity
Quercetin	Flavonol Catechol	Plant-derived natural product	Glucosides present in plant extracts that show VitP activity. Studied as a purified compound for it biological benefits
Taxifolin	Flavonol Catechol	Plant-derived natural product	Glucosides likely present in plant extracts that show VitP activity
Phlorizin	Dihydrochalcone	Plant-derived natural product	Likely present in plant extracts that show VitP activity
Catechin	Flavan-3-ol Catechol	Plant-derived natural product	Present in plant extracts that show VitP activity
Epicatechin-3-O-gallate	Flavan-3-ol Catechol Pyrogallol	Plant-derived natural product	Present in plant extracts that show VitP activity
Epigallocatechin-3-O-gallate	Flavan-3-ol Pyrogallol	Plant-derived natural product	Present in plant extracts that show VitP activity
Esculetin	Coumarin Catechol	Plant-derived natural product	Present in plant extracts that show VitP activity. Shows VitP activity as purified compound
Esculin	Coumarin	Plant-derived natural product	Present in plant extracts that show VitP activity. Shows VitP activity as purified compound
Catechol	Catechol	Degradation product of plant-derived natural products	Shows VitP activity as purified compound
Pyrogallol	Pyrogallol	Degradation product of plant-derived natural products	Shows VitP activity as purified compound
Leucocyanidol	Flavan-3,4-diol Catechol	Plant-derived natural product	Present in plant extracts that show VitP activity
Procyanidin B1	Flavan-3-ol Catechol	Plant-derived natural product	Present in plant extracts that show VitP activity

From a more general bioactivity perspective, the difficulty in discerning a specific biological VitP activity for a specific compound is a direct result of the behavior of many flavonoids in bioactivity assays that makes them “Invalid Metabolic Panaceas” (IMPs). Nevertheless, this apparent dilemma can also be understood as an outline of opportunities for future experiments that seek to connect a network of multiple phytochemicals with the multitude of biological effects that collectively constitute the VitP activity. The promiscuity and questionable validity of the biological effects assigned to highly prominent flavonoid IMPs has been recognized by Ingólfsson et al. (114) and is also reflected in the human protein-protein interactions that have recently been presented by do Valle et al. (115): studying associations between 65 IMPs and closely related compounds vs. 299 diseases, yielded 1,525 known and 17,910 unknown associations within the human interactome consisting of 17,651 proteins and 351,393 interactions. While the study recognized the known poor PK properties of these compounds, the cited contemporary approaches that use nanoparticles are not suitable to explain for enhancement of bioavailability in the context of food and vitamins/micronutrients contained therein.

In addition to seeking answers to the above pivotal questions, more general insights were gained from the study with regard to the overarching goals (see Motivation of This Study). Reinterpretation of previously-documented outcomes can inspire new directions for flavonoid/VitP/VitC research as summarized by the following points.

- Newly developed research hypotheses should take into account that multiple chemically related, yet distinct constituents are required to unfold an essential biological role.
- Low-abundance (“micro”) components are more challenging to work with, but fully valid as putative agents; they provide important opportunities for discovery, even in the presence of other known bioactive factors that might overshadow such “micronutrients.”
- Specific and distinctive chemical and biological terminology is essential, whereas blanket terminology (e.g., “antioxidant polyphenols”) tends to oversimplify and prevent progress.

By highlighting previously unrecognized connections between documented outcomes, naming known culprits, and outlining recent advances as well as remaining challenges equally, this study hopes to inspire future interdisciplinary research that ideally can clarify the nature of Vitamin P and advance it from its resilient last place in the vitamin alphabet.

## Author's Note

In 1960, my [DS] personal interest in this topic originated in the seminar class of Dr. Charles Schwartz of Southwestern Oklahoma State University, Weatherford, OK, in which I wrote an assigned report on vitamins. Obviously, that report and the attendant presentation were much too broad for a single 50-min seminar but provided an early introduction to the VitP literature.

## Author Contributions

DS and GP wrote the manuscript. DS, JF, JG, and GP conceived and organized the structure and wrote the first draft of the manuscript. JB, AB-R, and JM contributed to the critical revision of the paper. All authors approved the final manuscript for the publication.

## Funding

The authors gratefully acknowledge funding through grant U41 AT008706 [Center for Natural Products Technologies (CENAPT)] from NCCIH and ODS of the NIH.

## Conflict of Interest

The authors declare that the research was conducted in the absence of any commercial or financial relationships that could be construed as a potential conflict of interest.

## Publisher's Note

All claims expressed in this article are solely those of the authors and do not necessarily represent those of their affiliated organizations, or those of the publisher, the editors and the reviewers. Any product that may be evaluated in this article, or claim that may be made by its manufacturer, is not guaranteed or endorsed by the publisher.

## Abbreviations

IMPS: invalid/improbable/interfering metabolic panaceas
PAC: proanthocyanidin
OPAC: oligomeric proanthocyanidin
VitC: Vitamin C
VitP: Vitamin P.
