# Supplementary material for: Do Certain Flavonoid IMPS Have a Vital Function?
Source: Front Nutr. 2021 Dec 1;8:762753. doi: 10.3389/fnut.2021.762753 (PMC8672243; doi:10.3389/fnut.2021.762753)
Supplement: Supplementary file 1 [file Data_Sheet_1.PDF]

Do Certain Flavonoid IMPS Have a Vital Function?

David S. Seigler,[1] J. Brent Friesen,[2,3,4,5] Jonathan Bisson,[2,3,4] James G. Graham,[2,3,4] Ana Bedran-Russo,[3,6] James B. McAlpine,[2,3,4] and Guido F. Pauli[2,3,4]

[1]Department of Plant Biology, University of Illinois at Urbana Champaign, Urbana Champaign, IL, USA; [2]Center for Natural Products Technologies, [3]Pharmacognosy Institute, and [4]Department of Pharmaceutical Sciences, College of Pharmacy, University of Illinois at Chicago, Chicago, IL , USA; [5]Physical Sciences Department, Dominican University, Lake Forest, IL, USA; [6]Department of Restorative Dentistry, College of Dentistry, University of Illinois at Chicago, Chicago, IL, USA

SUPPLEMENTARY MATERIAL

Table of Contents

S1. What is a Flavonoid? page 2

S2. Structure Activity Relationships page 3

S3. What is “Citrin”? page 3

S4. Vitamin C-like Activity of Pycnogenol page 4

S5. Biological Targets of Vitamin C Metabolism page 6

S1. What is a Flavonoid?

Flavonoids are a large class of plant phenols, which are classified by the oxygenation and unsaturation pattern of their C-ring. This redox relationship between flavonoids also explains why these compounds are closely related in terms of both their biosynthesis and their metabolism. The main classes of flavonoids are:

- Acyclic forms: chalcones
- Saturated C-ring: flavanones (4-keto; hesperitin group), flavanols (3-OH; catechin group), flavanonols (3-OH, 4-keto; taxifolin group) flavandiol (leucocyanidol group)
- Unsaturated C-ring: flavones (apigenin group); flavonols (quercetin and kaempferol group).

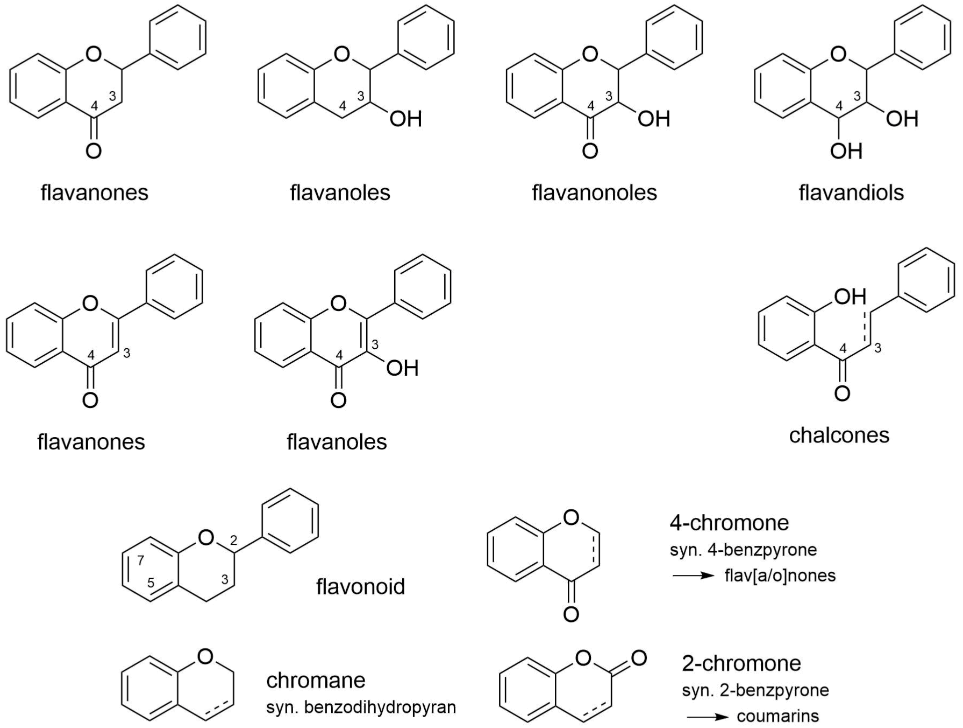

Figure S1. Structural similarities indicate shared biosynthetic and metabolic (ADME) pathways the various types of flavonoids, the chalcones and the coumarins. Also shown are the basic skeleton types of these ubiquitous phenolic plant constituents.

Flavonoids are exceedingly common in plants and, thus, a normal human diet. Flavonoids occur occasionally in other organisms, although their native biogenesis in most of these organisms is unconfirmed. Diverse structural types of flavonoids are known, with a major variation in the sugar moieties of flavonoid glycosides. Flavonoids play many roles in interactions within, between, and among organisms. They frequently serve as (co-)pigments in photosynthesis and are important in pollination as well as fruit and seed dissemination. They are found in several butterfly wings, where they influence recognition of prey by predators and identification of hosts or mates in the insects. Both flavonoids and tannins influence taste preference among animals including humans. Flavonoids are known to serve as both attractants and defensive compounds among insects. Some serve as signals for spore germination of fungi and are important in mycorrhizal interactions. Many of these highly diverse roles of flavonoids have been reviewed (1).

S2. Structure Activity Relationships

A number of compounds structurally similar to **1** were synthesized and tested for their structure activity relationships. The two enolic groups, D-configuration of carbon 4, and L-configuration of carbon 5 are necessary for maximum antiscorbutic activity (2,3). The primary terminal alcohol group also contributes significantly to the activity. Both the reducing and the antiscorbutic properties are lost when the lactone ring is opened (4).

S3. What is "Citrin"?

The term "citrin" has been used for two distinctively different entities (https://en.wikipedia.org/wiki/Citrin). On one hand, it refers to the Solute Carrier Protein (SLC), which is encoded by the human SLC25A13 gene (family 25, member 13). Citrin deficiency can lead to type II citrullinemia and neonatal intrahepatic cholestasis caused by citrin.

In the context of Vitamin P, citrin refers to the most active so-called bioflavonoid extract of lemons. Bruckner and Szent-Györgyi reported its chemical composition as hesperidine and eriodictyol glucoside in 1936 (5). Beyond this, a definitive chemical assignment cannot be made. A reasonable definition of citrin is *Citrus* flavonoids with Vitamin P bioactivity.

S4. What is Known about the Vitamin C-like Activity of Pycnogenol?

While lecturing in Quebec, Canada, Jack Masquelier learned that the famous explorer, Jacques Cartier, had an experience with scurvy. In the winter of 1534-1535, Cartier was caught in a sudden cold spell. A really disastrous winter during that time froze the St. Lawrence River and trapped Cartier and his crew of more than 100 men. Cartier's logbook described the horrible and awful details of how his men went into decay, weakened quickly, and died from scurvy. By chance, while trading with the natives, Cartier met an Indian who told him about a certain coniferous tree that could cure the dreaded disease from which his crewmen were suffering. Cartier found the tree, boiled the bark and the needles, made it into a tea — or extract — and gave it to his men. The scurvy from which they were suffering completely disappeared in many of them after as little as 48 hours (6,7).

Upon his return to France, Masquelier founded a company that prepared an extract of the bark from French maritime pine, *Pinus pinaster* Aiton *subsp. atlantica* Villar that he called pycnogenol (8). However, another company preempted him and trademarked that name for a similar extract in the United States. Nonetheless, Masquelier persisted and in the 1960s founded another company to manufacture a similar extract that is also marketed in the United States today under another name. An extract of pine bark, Pycnogenol®, contains 65-75 percent proanthocyanidins. Chemical identification studies showed that this extract is primarily composed of procyanidins that are biopolymers of catechin (**4**) and epicatechin (**5a**) with up to 7 or more flavonoid subunits and phenolic acids. The extract also contains monomeric catechin (**4**) and taxifolin (**3b**) (Figure 1). In humans, ferulic acid and **3b** components are rapidly absorbed and excreted as glucuronides or sulfates, whereas procyanidins are poorly absorbed as such, and are excreted or metabolized to valerolactone derivatives, which are excreted as glucuronides (9,10). More than 40 proanthocyanidins have been reported from Pycnogenol®. Thus, 100 mg would contain 65 to 75 mg of these substances. The pre-clinical and clinical aspects of Pycnogenol® have been carefully reviewed (8).

Needles and shoots from several *Pinus* and other gymnospermous species possess modest amounts of Vitamin C and have previously been used to alleviate the symptoms of scurvy during times of scarcity, such as the siege of Leningrad during World War II (11). Although pine needles and young shoots contain modest amounts of Vitamin C (11), bark extracts such as Pycnogenol® do not appear to contain Vitamin C. However, it has also been concluded that "current evidence is insufficient to support Pycnogenol® use for the treatment of any chronic disorder. Well-designed trials are needed to establish the value of this treatment" (12).

The question remains whether pine extracts represent a source of Vitamin C and accompanying Vitamin P compound(s), or if the extract can essentially replace a Vitamin C-rich preparation such as lemon juice. If, in fact, extracts which do not contain even a residual amount of Vitamin C can replace Vitamin C-rich extracts in scurvy treatment, then this discovery is revolutionary indeed and deserves follow-up studies. Despite its success, the difficulty of growing, harvesting, and processing materials from this *Pinus* species, the length of time needed to cultivate the trees, and the fact that harvest killed the trees, a more available source is favored.

Considering the evidence collected so far, and with Masquelier's Pycnogenol® and other oligomeric pro(antho)cyanidins OP(A)Cs (referred to as OPACs in the following), the Vitamin P hypothesis takes on a life of its own outside of its early postulated role as an enhancer of Vitamin C efficacy to treat scurvy. In addition some tests indicate specific biological targets for some Vitamin P constituents, and these are likely distinct from, or at least in addition to general antioxidant activity. In the case of Vitamin P, general health claims of a particular extract and/or formulation take precedence over the search for a single chemical entity with a specific biological target. In effect, residual complexity is not seen to present an analytical challenge for the creation of purified chemical entities but rather, it is embraced as an essential aspect of the therapeutic value. This approach is consistent with today's marketing strategy of many, if not all, dietary supplements of botanical origin. While this does not preclude the possibility of employing certain highly abundant flavonoids, as summarized above, as purified compounds in dietary supplements, strong mechanistic pre-clinical rationales that go beyond the early physiological studies summarized herein are still missing.

S5. Biological Targets of Vitamin C Metabolism

Vitamin C has important roles in several hydroxylases involved in the metabolism of neurotransmitters, steroids, drugs, and lipids. The content of **1** in the adrenal cortex is depleted rapidly when stimulated by the adrenocorticotropic hormone (ACTH), indicating that **1** plays a role in the synthesis and regulation of adrenalin and noradrenalin (4,13). It serves as an electron donor for a copper enzyme located in the chromaffin vesicles of the adrenal medulla and in adrenergic synapses, dopamine β-monooxygenase, which hydroxylates dopamine (**2a**) to form the neurotransmitter, norepinephrine (**2b**; syn. noradrenalin). In this reaction, ascorbate is oxidized to dehydroascorbate (**1b**), which is returned to the reduced state by monodehydroascorbate reductase and dehydroascorbate reductase (14).

The amino acids tyrosine and phenylalanine are not metabolized completely in Vitamin C-deficient individuals. Vitamin C appears to play a role as a cofactor in the metabolism of tyrosine (4). However, the best characterized metabolic role of **1** is in the synthesis of collagen proteins. The compound is involved in the hydroxylation of specific prolyl and lysyl residues of the unfolded (non-helical) procollagen chain, reactions that are catalyzed by the enzymes prolyl 4-hydroxylase, prolyl 3-hydroxylase, and lysyl hydroxylase. Each is a dioxygenase that requires O_2, Fe^{2+}, and **1**, while stoichiometrically linked to oxidative decarboxylation of α-ketoglutarate. It is thought that the role of **1** in each reaction is to maintain iron in the reduced state (Fe^{2+}), which dissociates from a thiol group in the active site to reactivate the enzyme after catalysis (14). The post-translational hydroxylation of these procollagen amino acid residues is necessary for folding into the triple helical structure that can be secreted by fibroblasts.

References

1. Seigler DS. Plant Secondary Metabolism [Internet]. Springer Science & Business Media; 2012. 759 p.

2. Reichstein T, Schwarz L, Grüssner A. Zur Kenntnis des Vitamnis C. Synthese der 6-Methyl-l-arabo-3-keto-hexonsäure-lactons (l-Rhamno-ascorbinsäure). Helv Chim Acta [Internet]. Wiley Online Library; 1935;18:353–4.

3. Reichstein T, Grüssner A, Oppenauer R. Synthese der d-und l-Ascorbinsäure (C-Vitamin). Helv Chim Acta [Internet]. Wiley Online Library; 1933;16:1019–33.

4. Charles O. Wilson, Ole Gisvold, J. B. Textbook of organic medicinal and pharmaceutical chemistry. 3rd ed. Lippincott Co., Philadelphia; 1956.

5. Bruckner V, Szent-Györgyi A. Chemical Nature of Citrin. Nature [Internet]. 1936;138:1057–1057.

6. Spounias J. The Mysterious "Vitamin P." The American Free Press [Internet]. 2015;

7. Schwitters B, Masquelier J. OPC in practice: Biflavanals and their application. Alfa Omega, Rome, Italy. 1993;

8. Oliff HS, Blumenthal. M. Scientific and Clinical Monograph for PYCNOGENOL® [Internet]. American Botanical Council; 2010.

9. Rohdewald P. Pleiotropic Effects of French Maritime Pine Bark Extract to Promote Healthy Aging. Rejuvenation Res [Internet]. 2019;22:210–7.

10. Rohdewald P. A review of the French maritime pine bark extract (Pycnogenol), a herbal medication with a diverse clinical pharmacology. Int J Clin Pharmacol Ther [Internet]. europepmc.org; 2002;40:158–68.

11. Raal A, Nisuma K, Meos A. Pinus sylvestris L. and other conifers as natural sources of ascorbic acid. Journal of Pharmacy & Pharmacognosy Research [Internet]. Asociación de Académicos de Ciencias Farmacéuticas de Antofagasta; 2018;6:89–95.

12. Schoonees A, Visser J, Musekiwa A, Volmink J. Pycnogenol(®) for the treatment of chronic disorders. Cochrane Database Syst Rev [Internet]. 2012;CD008294.

13. Hughes CD, Swenson MJ, Underbjerg GKL, Hughes JS. The Function of Vitamin C in the Adrenal Cortex [Internet]. Science. 1952. p. 252–3.

14. Combs GF, Jr. The Vitamins [Internet]. Academic Press; 2012. 598 p.
